# Catheter ablation and cognitive function in atrial fibrillation: A systematic review and meta-analysis

**DOI:** 10.3389/fneur.2022.934512

**Published:** 2022-09-08

**Authors:** Pengfei Chen, Zhuhong Chen, Deng Pan, Lina Miao, Yujiao Shi, Ming Guo, Jianpeng Du

**Affiliations:** ^1^Xiyuan Hospital, China Academy of Chinese Medical Sciences, Beijing, China; ^2^Cardiovascular Diseases Center, Xiyuan Hospital, China Academy of Chinese Medical Sciences, Beijing, China; ^3^Graduate School of Beijing University of Traditional Chinese Medicine, Beijing, China

**Keywords:** catheter ablation, cognitive function, dementia, atrial fibrillation, systematic review, meta-analysis

## Abstract

**Background:**

Atrial fibrillation (AF) is a risk factor for cognitive dysfunction. Although catheter ablation (CA) is one of the main treatments for AF, whether it can improve cognitive function in patients with AF remains unclear. We conducted a systematic review and meta-analysis to evaluate the cognitive outcome post-CA procedure.

**Methods:**

Two investigators independently searched the PubMed, EMBASE, Web of Science, CNKI, WanFang, and VIP databases from inception to September 2021 for all the potentially eligible studies. The outcomes of interest included dementia or cognitive disorder through scoring or recognized classification criteria. Heterogeneity was determined by using Cochrane's Q test and calculating the *I*^2^. A random-effects model was used to incorporate the potential effects of heterogeneity. The Newcastle-Ottawa Scale (NOS) was used to assess the methodological quality of each included study, and the Grading of Recommendations Assessment, Development and Evaluation (GRADE) method was adopted to evaluate the quality of evidence.

**Result:**

Thirteen studies including 40,868 patients were included, among which 12,086 patients received AF ablation. Meta-analysis indicated that patients with AF ablation had a lower risk of dementia incidence in comparison to patients with AF without ablation [hazard ratio (HR): 0.60, 95% CI: 0.43 to 0.84, *p* = 0.003 *I*^2^ = 40%]. Significant differences were observed in the incidence of new-onset dementia [risk ratio (RR): 0.43, 95% CI: 0.28 to 0.65, *p* < 0.0001 *I*^2^ = 84%]; the changes in the Montreal Cognitive Assessment (MoCA) score [weighted mean difference (WMD): 1.00, 95% CI: 0.36 to 1.64, *p* < 0.005 *I*^2^ = 0%] and Mini-Mental State Examination (MMSE) score (WMD: 0.98, 95% CI: 0.69 to 1.26, *p* < 0.00001 *I*^2^ = 0%]. However, in subgroup analysis, we did not observe significant changes in MoCA score at < 3 months (WMD: 1.20, 95% CI: −0.19 to 2.58, *p* = 0.09 *I*^2^ = 50%) and changes in cognitive function scores between the radiofrequency group and cryoballoon group [standard mean difference (SMD): 0.39, 95% CI: −0.47 to 1.24, *p* = 0.38 *I*^2^ = 87%]. The NOS indicated that included studies were moderate to high quality, while the quality of evidence assessed by GRADE was low in 2 and very low in 2.

**Conclusion:**

We analyzed the related cognitive outcomes after AF ablation. In the overall population, AF ablation had a positive trend for improving cognitive function at >3 months post-procedure. However, AF ablation might not be related to the improvement of cognitive function at < 3 months.

**Systematic review registration:**

https://www.crd.york.ac.uk/PROSPERO/, identifier: CRD42021285198.

## Introduction

Atrial fibrillation (AF) is the most common of all sustained arrhythmia with a worldwide prevalence of around 46.3 million individuals in 2016, the majority of whom are older adults (1, 2). Dementia is another major cause of morbidity in older adults, and more than 50 million people are living with dementia worldwide (3, 4). It is estimated that by 2050, as the population of the United States ages, the prevalence of AF and dementia will increase by between 2.5- and 3.0-fold (5).

There is increasing evidence pointing to dementia and cognitive disorder as additional adverse outcomes associated with AF. A recent meta-analysis showed that patients with AF had a 36% increased risk of developing dementia (6). An increased risk of stroke resulting from AF could partly mediate this association (7–9). Other adverse cerebrovascular effects associated with AF included cerebral hypoperfusion (10, 11), microbleeds (12, 13), and systemic inflammation (14), which might play a role but are not well characterized. The impact of cognitive dysfunction on healthcare and society will only increase along with the significant disease burden of AF.

Catheter ablation (CA) represents the first-line therapy for treating symptomatic and drug-refractory AF (15). In addition, a recent meta-analysis showed that CA as a first-line strategy in patients with paroxysmal AF had potential utility compared with anti-arrhythmic drugs (16). CA is superior to drug therapy in suppressing AF and improving symptoms, exercise capacity, and quality of life in patients with AF (17). The development and refinement of AF ablation have emerged as an effective therapy for AF and again raises the question of whether CA could attenuate a cognitive impairment. AF ablation on one side might reduce the risk of stroke, cerebral thromboembolism, and hypoperfusion with long-term sinus rhythm maintenance (18), and CA could also reduce the antiarrhythmic drug burdens used for rhythm control (19). However, on the other side, silent cerebral lesions (SCLs) during the AF ablation procedure might adversely increase the risk of post-procedural dementia (20). Consequently, there is a contradiction in the association of AF ablation with cognitive function. Therefore, we aimed to perform a systematic review and meta-analysis to evaluate the cognitive outcome post-CA procedure.

## Information and methods

### Research design and registration

Our systematic review and meta-analysis were reported according to the criteria outlined in the Meta-Analysis of Observational Studies in Epidemiology (MOOSE) and the PRISMA 2020 (21). This systematic evaluation program was registered in the PROSPERO International Prospective Registration for Systematic Evaluation (PROSPERO number: CRD42021285198).

### Data sources and search strategy

Two investigators (Peng-fei Chen and Deng Pan) independently and systematically searched the PubMed, EMBASE, Web of Science, CNKI, WanFang, and VIP databases from inception to 28 September 2021. The search MESH term and keywords used included “atrial fibrillation,” “catheter ablation,” “radiofrequency ablation,” “cryoablation,” “dementia,” “dementia, vascular,” “Alzheimer's disease,” “cognitive dysfunction,” “cognition disorder,” and “mental status test.” Detailed search strategies are shown in the Supplementary material. No restrictions on language, publication date, or publication status were set in our study. In addition, we examined the relevant reviews and reference lists of the included articles for further eligible studies. All the disagreements were resolved by consulting a third investigator (Ming Guo).

### Study selection

Two investigators independently screened titles, abstracts, and full-text material to select studies that met the following eligibility criteria: (1) all participants with AF (including permanent AF, persistent AF, and paroxysmal AF) are > 18 years old, human, and without a dementia history. (2) Studies that included a group of patients with AF treated with AF ablation (including radiofrequency (RF) and cryoballoon (CY) ablation). (3) Outcomes of interest should include dementia or cognitive disorder through scoring or recognized classification criteria. (4) Observational studies or clinical trials with at least 3 months of the follow-up period were considered for inclusion. The abstracts, editorial, animal experiment, or review were excluded.

### Data extraction

Prespecified data variables were extracted independently by two investigators. General characteristics included the author, year, country, study design, sample size of participants, follow-up duration in months, history of stroke, and maximum adjusted covariates. Baseline characteristics included demographic data (age and gender), combined diseases (hypertension, diabetes, and stroke/transient ischemic attack), combined drugs (anticoagulant and antiplatelet), and CHA2DS2-VASc score. Baseline characteristics of pooled study populations were reported as median values and their interquartile ranges (IQRs).

### Quality evaluation

The methodological quality of the included studies was assessed according to the Newcastle-Ottawa Scale (NOS) (22) with scores ranging from 0 to 9. We evaluated quality concerning patient selection, comparability of studies, and assessment of outcomes or exposures. Studies with a total NOS score of ≥8 stars were defined as high quality, NOS score of 6–8 stars as moderate quality, and NOS score of < 6 stars as low quality.

The Grading of Recommendations Assessment, Development and Evaluation (GRADE) method (23) was adopted to evaluate the quality of evidence. The GRADE working group rated the certainty of outcome evidence as high, moderate, low, or very low certainty of evidence based on the study design, risk of bias, inconsistency, indirectness, imprecision, and other considerations.

### Cognitive outcomes

The incidence of new-onset dementia including dementia Alzheimer's type, vascular dementia, senile dementia, frontotemporal dementia, dementia with Lewy bodies, and individual cognitive impairment reported in this study. The scales for evaluating cognitive function include Montreal Cognitive Assessment (MoCA) score, Mini-Mental State Examination (MMSE), and Telephone Interview for Cognitive Status-modified (TICS-m). The reliable change index was used to analyze the neuropsychological testing scores and to identify postoperative neurocognitive dysfunction (POCD).

### Statistical analysis

Hazard ratios (HRs) with 95% confidence intervals (CIs) for the incidence of dementia were extracted from published data. If adjustments were made for HRs, the most adequately adjusted HRs were extracted. For dichotomous variables, risk ratios (RRs) with 95% CIs were calculated. Continuous variables were calculated and expressed as weighted mean differences (WMDs) or standard mean differences (SMDs). Heterogeneity was assessed by using the Cochrane Q statistics, (*p* < 0.1 was considered with statistical heterogeneity), and I^2^ Statistics (25, 50, and 75% were considered to represent low, medium, and high heterogeneity, respectively). We adopted a random-effect model for the meta-analysis because it incorporates the potential effects of heterogeneity and therefore allows for the retrieval of more generalizable results. Sensitivity analyses by removing one individual study at a time to confirm the robustness of the results. All statistical analyses were carried out using the Review Manager 5.4 software.

## Results

### Study search

**Figure 1:**
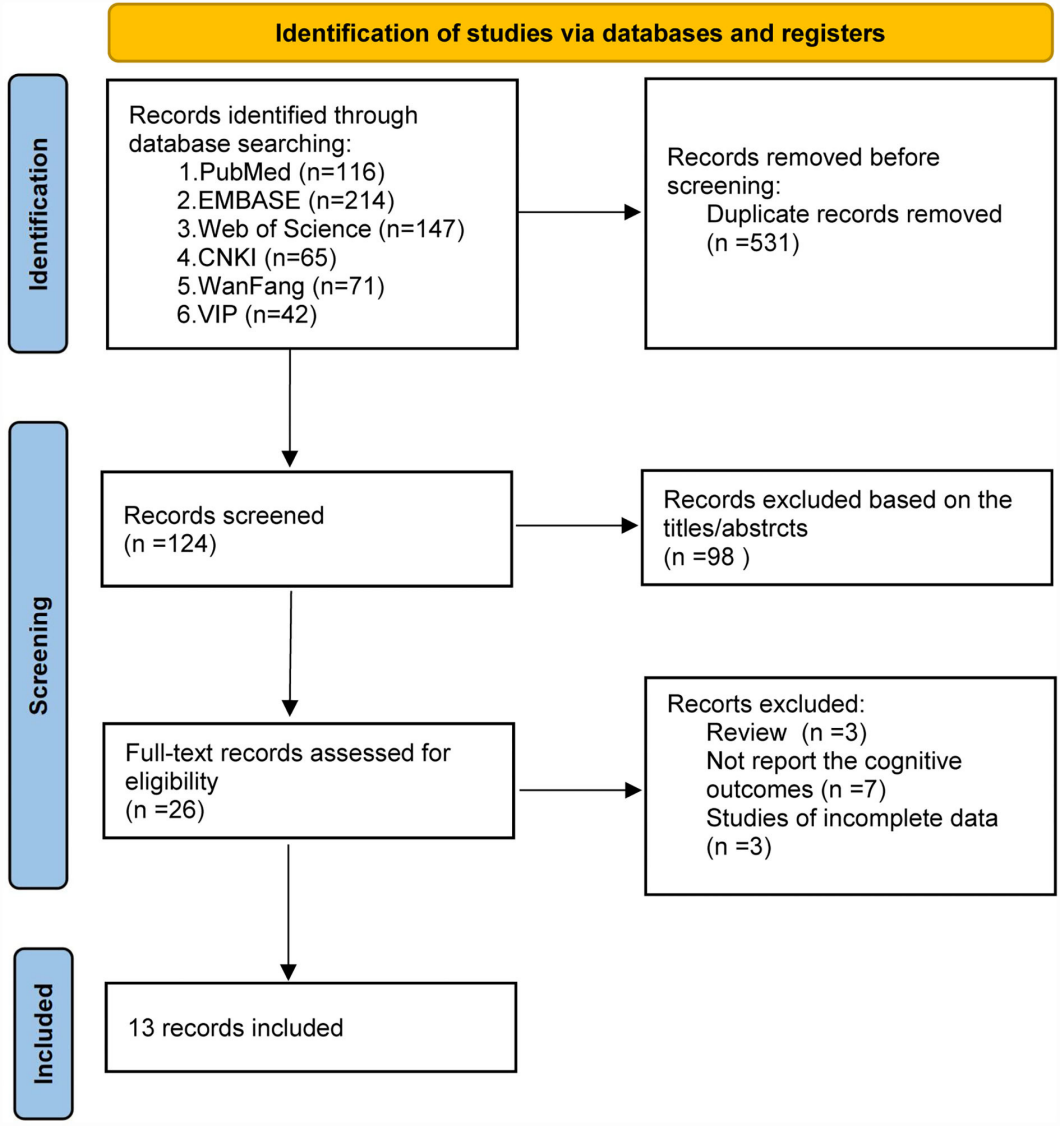
Flow diagram of study selection and identification.

The process of the database search and study identification is presented in Figure 1. A total of 655 records were retrieved from 6 databases, 531 were duplicates, and 98 studies were excluded based on title and abstract primarily because they were irrelevant to the study purpose. The remaining 26 articles were evaluated for eligibility by full-text screening. Of these, 13 studies were further excluded because 3 studies were reviews, 7 studies did not report the cognitive outcomes, and the other 3 were studies of incomplete data. Finally, 13 studies (24–36) were included in our systematic review and meta-analysis.

### Study characteristics

Table 1 displays the general characteristics, comprising 40,868 individuals (12,086 patients with AF ablation) included in the meta-analysis. The median follow-up time ranged from 3 months to 9 years. In this systematic review and meta-analysis, 4 studies (24–27) were retrospective cohort studies and 9 (28–36) were controlled clinical trials. Six studies (26, 30–32, 34, 35) were conducted in China, 2 (24, 29) in South Korean, 2 (25, 27) in the United States, 1 (28) in Germany, 1 (36) in Japan, and 1 (33) in Australia. Four studies (24–27) reported the incidence of new-onset dementia, 4 (28–31) reported the changes in MoCA score, 3 (28, 35, 36) reported the MMSE score, 1 (32) reported the TICS-m score, and 2 (33, 34) reported the incidence of POCD.

**Table 1:** General characteristics of the included studies.

**References**	**Year**	**Country**	**Study Design**	**Follow up**	**CA Patients**	**Patients**	**Stroke Exclusion**	**Variables adjusted**
Kim et al. (24)	2020	South Korea	Re cohort	52 months	5,863	1,1726	no	Socio-demographics, clinical risk scores,medical history, drug treatments for AF, concurrent medication use, AF duration.
Bunch et al. (25)	2019	United State	Re cohort	70 mouths	442	5,549	no	Age, hypertension, diabetes, hyperlipidemia, renal failure, smoking history, prior myocar-dial infarction or cerebral vascular accident, heart failure.
Hsieh et al. (26)	2019	Taiwan	Re cohort	108 months	787	1,574	no	Age, gender, hypertension, chronic kidney disease, chronic obstructive pulmonary disease.
Bunch et al. (27)	2011	United State	Re cohort	60 mouths	4,212	21,060	no	-
Tischer et al. (28)	2019	Germany	Controlled clinical trials	6 months	12	21	yes	-
Tischer et al. (28)	2019	Germany	Controlled clinical trials	6 months	16	24	yes	-
Jin et al. (29)	2019	South Korean	Controlled clinical trials	12 months	150	200	no	Age, gender, prior stroke/transient ischemic attack, baseline MoCA score.
Huang et al. (30)	2021	China	Controlled clinical trials	6 months	34	73	yes	-
Xia et al. (31)	2014	China	controlled clinical trials	3 months	30	60	no	-
Wang et al. (32)	2021	China	Controlled clinical trials	12 months	98	139	yes	-
Medi et al. (33)	2013	Australia	Controlled clinical trials	6 months	90	90	no	-
Zhang et al. (34)	2021	China	Controlled clinical trials	6 months	190	190	yes	-
Li et al. (35)	2020	China	Controlled clinical trials	6 months	88	88	yes	-
Kato et al. (36)	2021	Japan	Controlled clinical trials	6 months	74	74	no	-

Table 2 displays the baseline characteristics. AF with ablation group compared with AF without ablation group, the median age was 60.1 years (IQR 57.6–66.3 years)/64.5 years (IQR 59.5–67.5 years), median patients of male were (70%, IQR 52.6–75.3%)/(56.7%, IQR 40.1–2.4%), median patients of hypertension were (48.9%, IQR 43.5–68.5%)/(53.5%, IQR 38.9–47.2%), and median patients of diabetes were (18.1%, IQR 16.4–21.2%)/(23.4%, IQR 18.3–28.4%). The median number of patients with a history of previous stroke and/or transient ischemic attack was 8.4% (IQR 0–12.8%)/3.6% (IQR 0–13.7%). The median CHA2DS2-VASc score was 1.6 (IQR 1.2–2.2)/2 (IQR 1.5–2.9), with 64.8% (IQR 53.1–100%)/56.9% (IQR 34.7–100%) of patients on oral anticoagulant and 36.7% (IQR 13.6–77.4%)/33.3% (IQR 29.3–65.6%) of patients on oral antiplatelet. Four studies (33–36) lacked the comparability of other studies due to their methods of recruiting consecutive patients awaiting CA.

**Table 2:** Baseline characteristics of the included studies.

**References**	**Age(O/C)**	**Male(O/C)(%)**	**Hypertension(O/C)(%)**	**Diabetes(O/C)(%)**	**Stroke/TIA(O/C)(%)**	**Anticoagulant(O/C)(%)**	**Antiplatelet(O/C)(%)**	**CHA2DS2-VASc Score (Median)(O/C)**
Kim et al. (24)	60/60	74.1/74.8	80.4/81.1	17.8/17.7	30.3/30.3	64.8/64.7	27.1/26.8	2/2
Bunch et al. (25)	73.7/73.5	24.2/17.3	74.7/78.6	21.3/29	16.4/16.9	56/34.7	58/47.2	4.5/4.5
Hsieh (26)	54.1/54.9	70.1/70	36.8/36.8	8/6.7	8.4/3.6	37/56.9	96.7/84	1/0
Bunch et al. (27)	64.8/66	60.8/60.8	47.8/45.3	16.3/21.1	9.1/10.5	–	–	–
Tischer et al. (28)	67.8/67.8	44.4/28.9	–	–	0/0	–	–	–
Jin et al. (29)	60.1/60.3	78.7/78	50/48	20.7/20	8.7/8	100/100	–	1.6/1.5
Huang et al. (30)	64.7/67.2	67.6/51.3	44.1/59	29.4/25.6	0/0	100/100	–	2.2/2.9
Xia et al. (31)	55.5/58.9	70/56.7	43.3/36.7	16.7/26.7	6.7/3.3	100/23.3	0/33.3	1.2/1.6
Wang et al. (32)	59.8/64.5	76.5/53.7	50/61	18.4/34.2	0/0	53.1/51.2	36.7/31.7	1.5/2
Medi et al. (33)	55	81.1	45.6	6.7	22.2	48.9	41.1	0.73
Zhang et al. (34)	66.6	59.5	64.2	17.4	0	61.1	10	–
Li et al. (35)	63	61.4	48.9	19.3	0	–	–	1.78
Katoet al. (36)	68.3	71.6	51.4	13.5	6.8	70.2	–	–

O, AF with ablation group; C, AF wituout ablation group; TIA, transient ischemic attack.

### Study quality

The NOS showed that the quality scores of all the included studies ranged from 6 to 9 (mean score: 7.3), indicating moderate to high quality. Table 3 shows the study quality.

**Table 3:** Quality assessment of the included studies.

**References**	**Select**				**Comparability**	**Outcome**			**Score**
	**Exposed Cohort**	**Nonexposed Cohort**	**Ascertainment of Exposure**	**Outcome of Interest**		**Assessment of Outcome**	**Length of Follow-up**	**Adequacy of Follow-up**	
Kim et al. (24)	^*^	^*^	^*^	^*^	**	^*^	^*^	^*^	9
Bunch et al. (25)	^*^	^*^	^*^		**	^*^	^*^	^*^	8
Hsieh et al. (26)	^*^	^*^	^*^	^*^	**	^*^	^*^	^*^	9
Bunch et al. (27)	^*^	^*^	^*^	^*^	^*^	^*^	^*^	^*^	8
Tischer et al. (28)	^*^	^*^	^*^	^*^	^*^	^*^		^*^	7
Jin et al. (29)	^*^	^*^	^*^	^*^	^*^	^*^	^*^	^*^	8
Huang et al. (30)	^*^	^*^	^*^	^*^	^*^	^*^		^*^	7
Xia et al. (31)	^*^	^*^	^*^	^*^	^*^	^*^		^*^	7
Wang et al. (32)	^*^	^*^	^*^	^*^	^*^	^*^	^*^	^*^	8
Medi et al. (33)	^*^		^*^	^*^		^*^	^*^	^*^	6
Zhang et al. (34)	^*^		^*^	^*^		^*^	^*^	^*^	6
Li et al. (35)	^*^		^*^	^*^		^*^	^*^	^*^	6
Kato et al. (36)	^*^		^*^	^*^		^*^	^*^	^*^	6

* represent stars used in the Newcastle Ottawa Scale.

Among these outcome indicators, the quality of evidence was low in 2 and very low in 2. Certainty assessment ratings and the summary of findings are presented in Table 4.

**Table 4:** Cognitive outcomes and GRADE classification in meta-analysis of the included studies.

**No of studies**	**Certainty assessment**						**Effect**			**Certainty**	**Importance**
	**Study design**	**Risk of bias**	**Inconsistency**	**Indirectness**	**Imprecision**	**Other considerations**	**No of AF ablation**	**No of not AF ablation**	**Relative (95% CI)**		
**Hazard Ratio of New-onset Dementia**											
3	Observational studies	Not serious^a^	Not serious^b^	Not serious^d^	Not serious^f^	Publication bias strongly suspected^g^ all plausible residual confounding would reduce the demonstrated effect^h^	7,092	11,757	HR 0.60 (0.43 to 0.84)	⊕⊕○○ Low	Crucial
**Incidence of New-onset Dementia**											
4	Observational studies	Not serious^a^	Not serious^b^	Serious^e^	Not serious^f^	publication bias strongly suspected^g^ all plausible residual confounding would reduce the demonstrated effect^h^	11,304	2, 8,605	RR: 0.43 (0.28 to 0.65)	⊕○○○ Very Low	Important
**MoCA score**											
4	Observational studies	Not serious^a^	Serious^c^	Not serious^d^	Not serious^f^	Publication bias strongly suspected^g^ all plausible residual confounding would reduce the demonstrated effect^h^	242	136	WMD: 1.00 (0.36 to 1.64)	⊕○○○ Very Low	Crucial
**MMSE score**											
3	Observational studies	Not serious^a^	Not serious^b^	Not serious^d^	Not serious^f^	Publication bias strongly suspected^g^ all plausible residual confounding would reduce the demonstrated effect^h^	178	178	WMD: 0.98 (0.69 to 1.26)	⊕⊕○○ Low	Crucial

a Risk of bias by NOS was judged low for individual studies (see Table 2).

b Appropriate population generalizability and outcomes applicability.

c The score was downgraded because the proportion of patients in jin's study is too high (weigh>50%).

d The heterogeneity was considered to represent low.

e The score was downgraded because substantial heterogeneity between studies was detected (I^2^>75%).

f Narrow 95% confidence interval.

g The score was downgraded because fewer studies were included and there may have been greater publication bias.

h The score was downgraded because all included studies in this meta-analysis were observational studies, we cannot rule out that some residual factors may reduce the demonstrated effect.

CI confdence interval, HR hazard ratio, RR risk ratio, WMD weighted mean difference, SMD standardized mean difference, AF atrial fibrillation.

### Results of the meta-analysis

#### New-onset dementia

**Figure 2:**
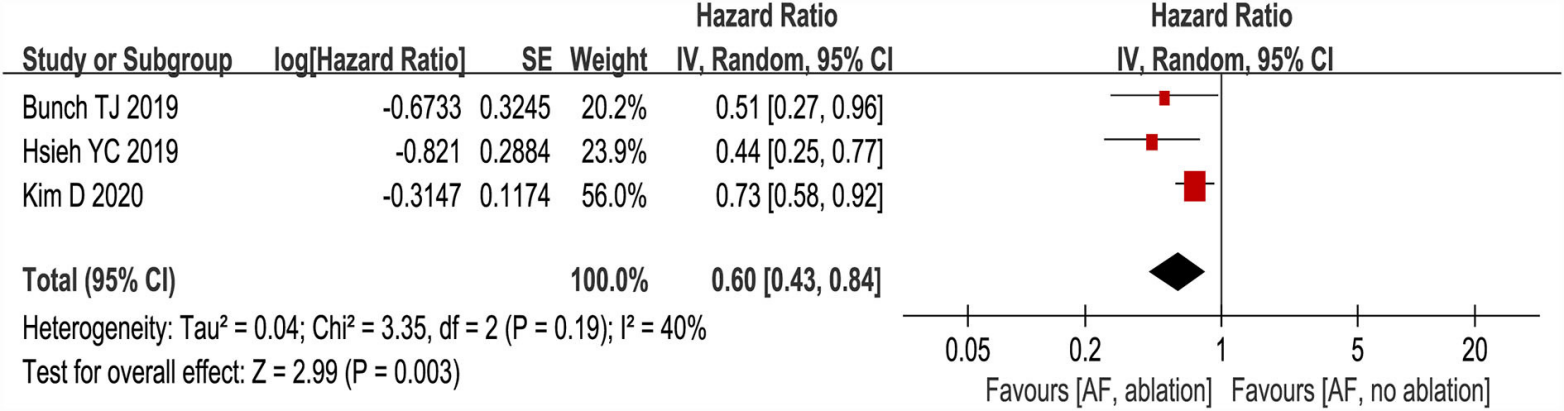
Forest plot present the meta-analysis for the association between the risk of dementia incidence and AF ablation.

**Figure 3:**
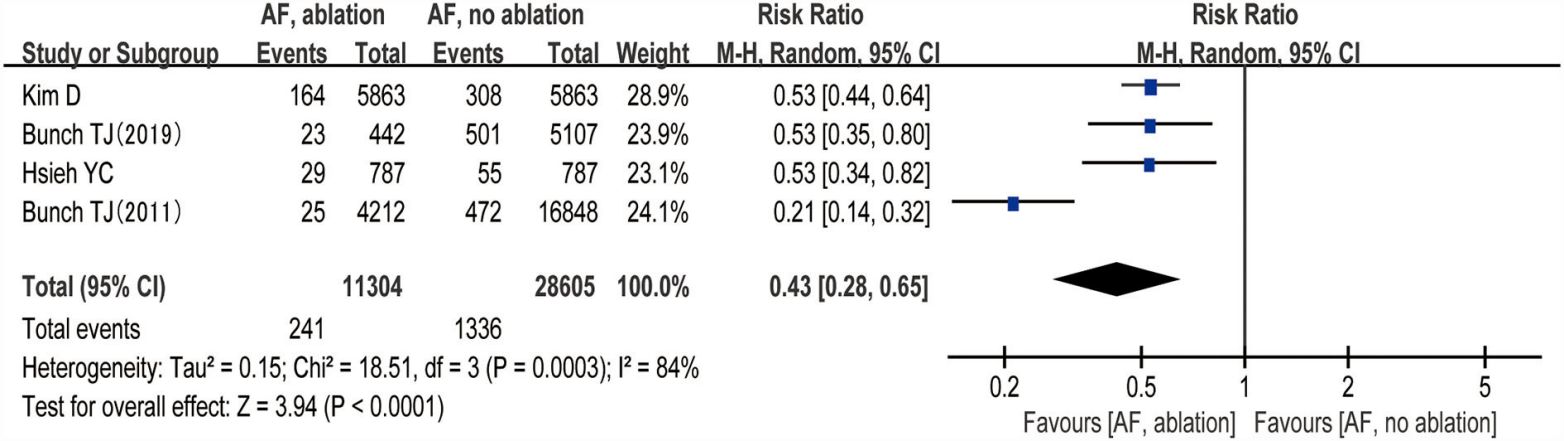
Forest plot present the meta-analysis for the incidence rate of dementia comparing AF with ablation vs. AF without ablation groups.

Three studies (24–26) evaluated the HRs of developing dementia, including 18,849 patients. We adopted a random-effects model to perform the meta-analysis, and the overall adjusted pooled HR of developing dementia was 0.60 (95% CI: 0.43 to 0.84, *p* = 0.003 *I*^2^ = 40%; Figure 2), which showed that patients with AF ablation compared with patients with AF without ablation had a 40% lower risk of developing dementia during follow-up. The sensitivity analysis results were consistent (HR: 0.47 to 0.69, *p* all < 0.05). We also conducted a meta-analysis of 4 studies (24–27) by dichotomous variables (random-effect RR: 0.43, 95% CI: 0.28 to 0.65, *p* < 0.0001 *I*^2^ = 84%; Figure 3). The sensitivity analysis results were consistent (RR: 0.39 to 0.53, *p* all < 0.05).

#### MoCA score

**Figure 4:**
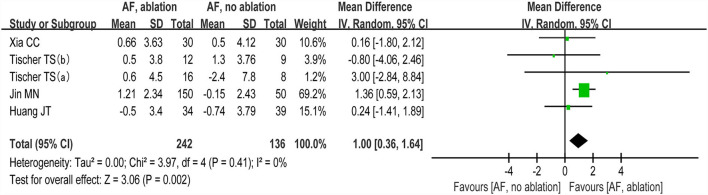
Forest plot present the meta-analysis for the changes of MoCA score comparing AF with ablation vs. AF without ablation groups.

The changes from the baseline of the MoCA score were reported in 4 studies (5 analyses) (27–30). A significant improvement of the MoCA score was identified between the AF with ablation group and AF without ablation group, which favored the AF with ablation group (random-effect WMD: 1.00, 95% CI: 0.36–1.64, *p* = 0.002 *I*^2^ = 0%; Figure 4). The sensitivity analysis results were consistent (WMD: 0.19–1.13). However, no statistical difference was found after removing the study of Jin et al. (WMD: 0.19, 95% CI: −0.96 to 1.35, *p* > 0.05). Notably, the number of patients included in the Jin et al. study was much higher than in other studies.

#### MMSE score

**Figure 5:**
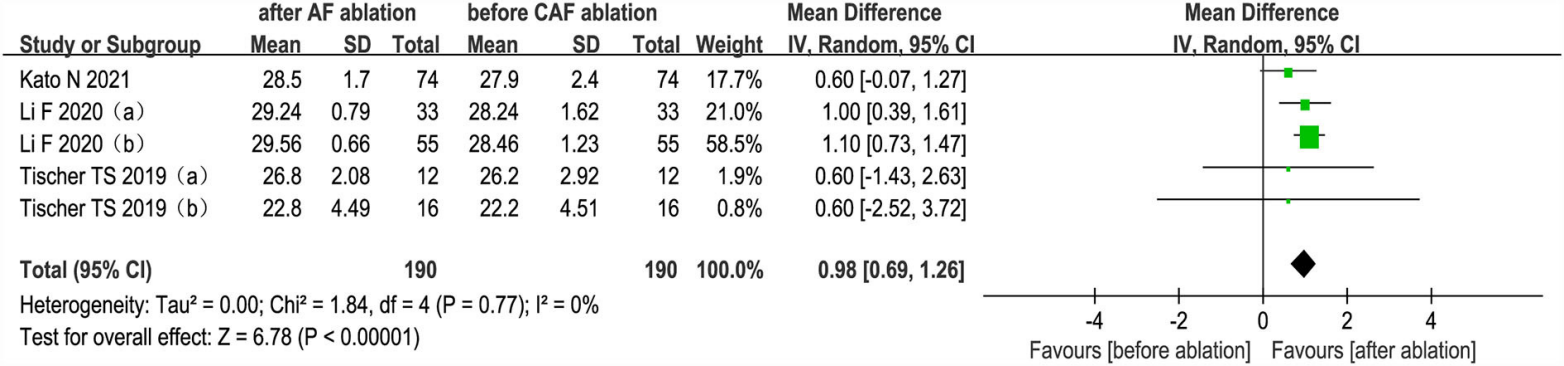
Forest plot present the meta-analysis for the changes of MMSE score comparing before AF ablation vs. after AF ablation groups.

The changes from the baseline of MMSE score were reported in 3 studies (5 analyses) (27, 35, 36). The MMSE score after AF ablation was significantly improved than before AF ablation (random-effect WMD: 0.98, 95% CI: 0.69 to 1.26, *p* < 0.00001 *I*^2^ = 0%; Figure 5). The sensitivity analysis results were consistent (SMD: 0.80 to 1.06, *p* all < 0.05).

#### TICS-m score

One study (31) that included 139 patients compared changes in TICS-m score in the AF ablation group and drug treatment group. The result indicated that TICS-m scores in the AF ablation group (39.56 ± 3.198) were significantly improved compared with the drug treatment group (34.44 ± 3.271) (*p* < 0.00001) at 12-month follow-up.

#### Incidence of POCD

Two studies (33, 34) evaluated the prevalence rates of POCD. Medi et al. found that the prevalence of POCD at 2-day post-procedure was 28% (17/60) in patients with paroxysmal AF (PAF) and 27% (8/30) in patients with persistent AF (PeAF). At 90-day follow-up, the prevalence was 13% (8/60) in PAF and 20% (6/30) in PeAF. Zhang et al. found that 13.7% (26/190) of patients with AF had POCD 2 days after post-procedure, and the global cognitive scores decreased 2 days after postoperation tests and improved significantly at 6 months postoperation. These two studies suggested that the higher incidence of POCD 2-day post-procedure may in part reflect the reversible effect of anesthesia on cognitive function. At long-time follow-up, AF ablation might be associated with cognitive function improvement.

#### Subgroup analysis

##### Follow-up time

**Figure 6:**
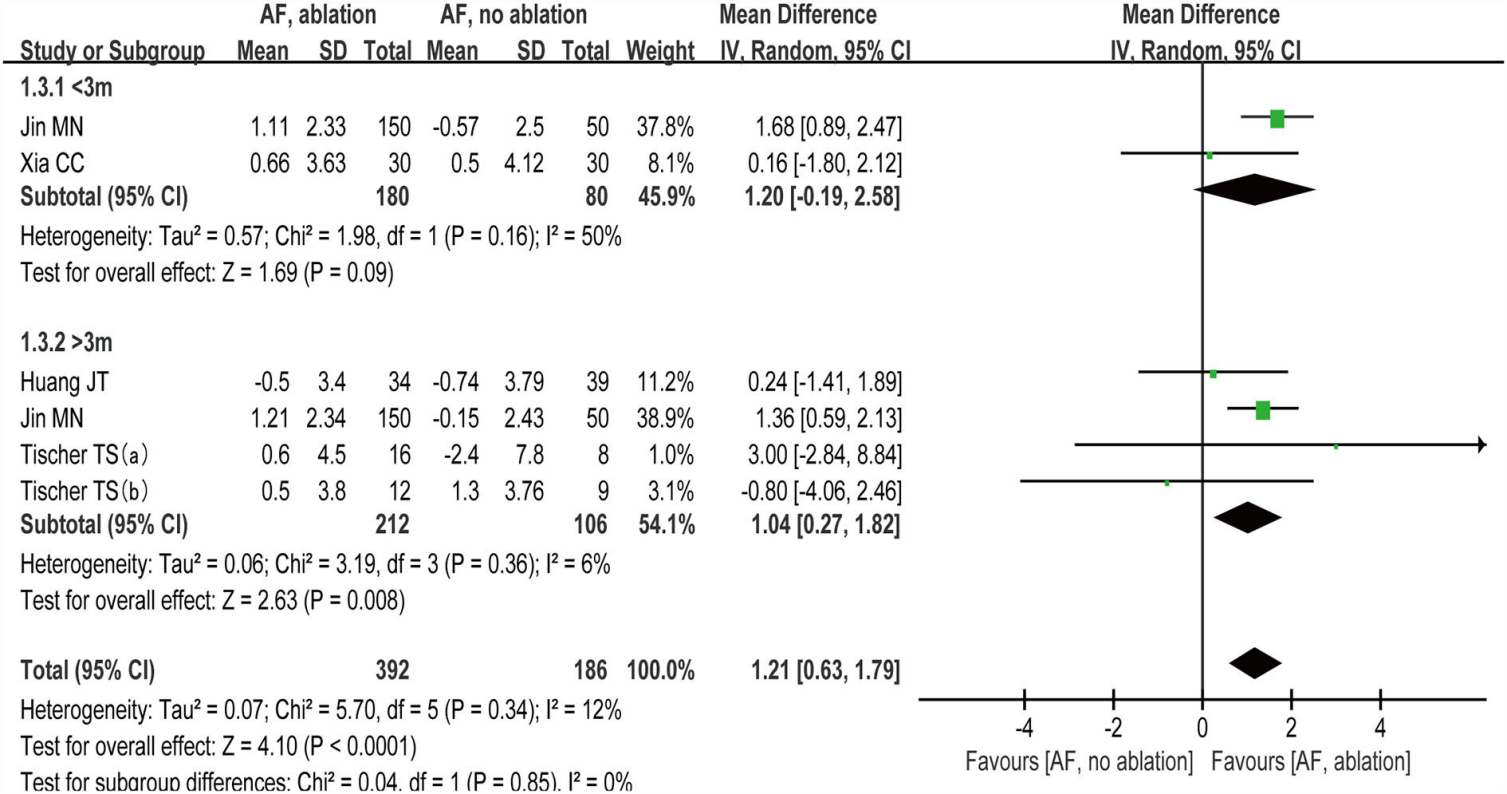
Forest plot present the subgroup analysis for the changes of MoCA score between the follow-up time with < 3 months and > 3 months.

We grouped studies (27–30) that reported MoCA scores by follow-up time with < 3 months and >3 months. Subgroup analysis showed (random-effect WMD: 1.20, 95% CI: −0.19 to 2.58, *p* = 0.09 *I*^2^ = 50%; Figure 6) at < 3 months and (random-effect WMD: 1.04, 95% CI: 0.27 to 1.82, *p*=0.008 *I*^2^= 6%; Figure 6) at > 3 months. There were no significant statistical differences at the < 3 month subgroup (*p* >0.05).

##### Radiofrequency and CY ablation

**Figure 7:**
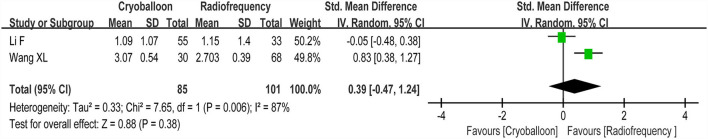
Forest plot present the subgroup analysis for the changes of cognitive function score between the RF group and the CY group.

Two studies reported changes in cognitive function scores between the RF group and the CY group. We found no statistically significant differences (*p*>0.05) in cognitive function scores between the RF group and CY group (random-effect SMD: 0.39, 95% CI: −0.47 to 1.24, *p* = 0.38, *I*^2^ = 87%; Figure 7).

## Discussion

This meta-analysis and systematic review of 13 studies examined the relationship between AF ablation and cognitive function. We found that patients with AF ablation were associated with a lower risk of developing dementia in comparison with patients without AF ablation. Moreover, AF ablation had a positive trend for improving cognitive evaluation scales as a whole. MoCA, MMSE, and TICS-m are common indicators for evaluating cognitive function. A significant improvement in the MoCA, MMSE, and TICS-m scores was identified. In the subgroup analysis, no significant statistical differences were found in changes in MoCA score at < 3 months and changes in cognitive function scores between the RF and CY group. Of note, 2 studies reported the prevalence rate of POCD. They compared post-procedural cognitive function at 2 days and more than 3 months with cognitive function immediately before AF ablation. Cognitive impairment seemed to be only temporary 2 days after the post-procedure, and late cognitive function improvement may be related to time-dependent improvement.

Previous studies had reported that AF ablation was associated with cognitive decline and acute brain lesions. A small prospective study enrolling 23 patients showed worse neuropsychological outcomes in verbal memory in the AF ablation group (37). Previously, new SCLs detected after CA were a common occurrence in magnetic resonance imaging (MRI) of the brain and were reported in 4.3–38.9% of patients (32, 38, 39). However, during MRI follow-up of more than 90% of patients 1 year after AF ablation, SCLs had been proven to be resolved (40, 41). The neurological impact of these SCLs is unclear and is likely to be determined by their size, number, and anatomic region. The sequelae of SCLs may include subtle neurocognitive impairment, which is in turn associated with an increased lifetime risk of cognitive impairment. Several aspects of the AF ablation may lead to acute cerebral injury and post-procedural cognitive dysfunction, for example, periprocedural thrombus, cerebral hypoperfusion, and anesthesia (42). Besides, catheters are a source of embolization, such as air embolization and carbonization, which may increase the risk of thromboembolic events, and lead to cognitive impairment (43).

In contrast to early observational studies, other emerging research had shown that AF ablation might improve cognitive function by reducing the AF burden and restoring sinus rhythm (44–46). Jin's study (29) suggested that AF ablation could reduce the possibility of left atrial thrombosis caused by atrial asynchronism and hemodynamic changes by relieving clinical symptoms and maintaining sinus rhythm in patients, thus improving long-term cognitive function. A recent randomized controlled trial (47) by Haeusler et al. has reported that chronic white matter damage and acute ischemic lesions detected by MRI were found frequently after first-time CA for paroxysmal AF using uninterrupted oral anticoagulation. The median of MoCA was similar in patients with or without acute brain damage at 3 months after CA, and acute ischemic lesions detected by high-resolution diffusion-weighted imaging were not associated with cognitive function at 3 months after ablation. We inferred that the restoration and maintenance of sinus rhythm were an important mechanism, rather than the AF ablation itself, which has been associated with at least transient worsening of cognitive function. The recovery of sinus rhythm after AF ablation improved atrioventricular synchronization and systolic and diastolic function, which may enhance cerebral perfusion, promoting the recovery and improvement of cognitive function.

Two previous meta-analyses also examined the relationship between AF ablation and dementia. A meta-analysis of 4 studies by Saglietto et al. (48) showed that AF ablation is associated with a nearly 50% reduction in dementia occurrence. Another meta-analysis by Bodagh et al. (49) found that AF ablation was associated with a lower risk of subsequent dementia diagnosis. However, the above two meta-analyses did not include sufficient studies and did not analyze other cognitive outcome indicators except dementia. Our study included all the current studies on the relationship between AF ablation and cognitive outcomes. We analyzed more comprehensive cognitive outcome indicators and conducted a subgroup analysis on follow-up time and ablation type, which provided more evidence information.

The advantages of our meta-analysis may include the following. First, the results of this study were relatively stable and reliable because the meta-analysis covered studies from different countries and had a large sample size. Second, only cohort studies and controlled clinical trials were included, and the results showed a sequential association between AF ablation and improvement in cognitive function. Third, the most adequately adjusted HRs were extracted, which reduced clinical heterogeneity to a certain extent. Fourth, the sensitivity analyses that omitted a study at a time had no significant impact on the results, suggesting that the outcomes were credible. Fifth, subgroup analyses of follow-up time and ablation type were conducted to assess the potential study characteristics of the relationship between AF ablation and cognitive function. Finally, the NOS was used to assess the methodological quality of the studies, and the GRADE method was adopted to evaluate the quality of evidence.

However, this meta-analysis also had some limitations. First, as a meta-analysis of observational studies, we were unable to determine whether the association between AF ablation and dementia was causal. Second, we cannot exclude that some residual factors may confound the association between AF ablation and cognitive function improvement, although we included studies with multivariate-adjusted HRs only. Third, in the real world, various drugs are commonly used to treat AF. Most of the included studies did not mention specific treatment regimens, which to some extent leads to an unavoidable clinical heterogeneity.

## Conclusion

We analyzed the related cognitive outcome post-CA procedure. In the overall population, AF ablation had a positive trend for declining the risk of developing dementia and improving cognitive function at >3 months post-procedure. However, AF ablation might not be related to the improvement of cognitive function at < 3 months and changes in cognitive function scores between the RF group and the CY group.

## Data availability statement

The original contributions presented in the study are included in the article/Supplementary material, further inquiries can be directed to the corresponding author.

## Author contributions

JD: conceptualization and supervision. PC and DP: data curation, formal analysis, investigation, and methodology. PC and LM: software and writing—original draft. ZC: drew and revised pictures. JD and MG: writing—review and editing and modifying the final version. All authors contributed to this study and approved the submitted version.

## Funding

This study was supported by the project of Major New Drug Creation (No. 2018ZX09301-011-001) and the National Natural Science Foundation of China (Grant No. 81904025).

## Conflict of interest

The authors declare that the research was conducted in the absence of any commercial or financial relationships that could be construed as a potential conflict of interest.

## Publisher's note

All claims expressed in this article are solely those of the authors and do not necessarily represent those of their affiliated organizations, or those of the publisher, the editors and the reviewers. Any product that may be evaluated in this article, or claim that may be made by its manufacturer, is not guaranteed or endorsed by the publisher.

## References

[1] Benjamin EJ Muntner P Alonso A Bittencourt MS Callaway CW Carson AP . Heart disease and stroke statistics-2019 update: a report from the American Heart Association. Circulation. (2019) 139:e56–e528. 10.1161/CIR.0000000000000659 30700139

[2] Lippi G Sanchis-Gomar F Cervellin G. Global epidemiology of atrial fibrillation: an increasing epidemic and public health challenge. Int J Stroke. (2021) 16:217–21. 10.1177/1747493019897870 31955707

[3] Grande G Qiu C Fratiglioni L. Prevention of dementia in an ageing world: evidence and biological rationale. Ageing Res Rev. (2020) 64:101045. 10.1016/j.arr.2020.101045 32171784

[4] Aarsland D. Epidemiology and pathophysiology of dementia-related psychosis. J Clin Psychiatry. (2020) 81:AD19038BR1C. 10.4088/JCP.AD19038BR1C 32936544

[5] Go AS Hylek EM Phillips KA Chang Y Henault LE Selby JV . Prevalence of diagnosed atrial fibrillation in adults: national implications for rhythm management and stroke prevention: the AnTicoagulation and Risk Factors in Atrial Fibrillation (ATRIA) Study. JAMA. (2001) 285:2370–5. 10.1001/jama.285.18.2370 11343485

[6] Islam MM Poly TN Walther BA Yang HC Wu CC Lin MC . Association between atrial fibrillation and dementia: a meta-analysis. Front Aging Neurosci. (2019) 11:305. 10.3389/fnagi.2019.00305 31780919 PMC6857071

[7] Mijajlović MD Pavlović A Brainin M Heiss WD Quinn TJ Ihle-Hansen HB . Post stroke dementia - a comprehensive review. BMC Med. (2017) 15:11. 10.1186/s12916-017-0779-7 28095900 PMC5241961

[8] Lip G Freedman B De Caterina R Potpara TS. Stroke prevention in atrial fibrillation: Past, present and future. Comparing the guidelines and practical decision-making. Thromb Haemost. (2017) 117:1230–9. 10.1160/TH16-11-0876 28597905

[9] Hachinski V Einhäupl K Ganten D Alladi S Brayne C Stephan BCM . Preventing dementia by preventing stroke: the berlin manifesto. Alzheimers Dement. (2019) 15:961–84. 10.1016/j.jalz.2019.06.001 31327392 PMC7001744

[10] Anselmino M Scarsoglio S Saglietto A Gaita F Ridolfi L. Transient cerebral hypoperfusion and hypertensive events during atrial fibrillation: a plausible mechanism for cognitive impairment. Sci Rep. (2016) 6:28635. 10.1038/srep28635 27334559 PMC4917883

[11] Daulatzai MA. Cerebral hypoperfusion and glucose hypometabolism: key pathophysiological modulators promote neurodegeneration, cognitive impairment, and Alzheimer's disease. J Neurosci Res. (2017) 95:943–72. 10.1002/jnr.23777 27350397

[12] Cannistraro RJ Badi M Eidelman BH Dickson DW Middlebrooks EH Meschia JF. CNS small vessel disease: a clinical review. Neurology. (2019) 92:1146–56. 10.1212/WNL.0000000000007654 31142635 PMC6598791

[13] Lim EY Ryu SY Shim YS Yang DW Cho AH. Coexistence of cerebral microbleeds and amyloid pathology in patients with cognitive complaints. J Clin Neurol. (2020) 16:83–89. 10.3988/jcn.2020.16.1.83 31942762 PMC6974843

[14] Chojdak-Łukasiewicz J Dziadkowiak E Zimny A Paradowski B. Cerebral small vessel disease: a review. Adv Clin Exp Med. (2021) 30:349–56. 10.17219/acem/131216 33768739

[15] Buist TJ Zipes DP Elvan A. Atrial fibrillation ablation strategies and technologies: past, present, and future. Clin Res Cardiol. (2021) 110:775–88. 10.1007/s00392-020-01751-5 33089361

[16] Turagam MK Musikantow D Whang W Koruth JS Miller MA Langan MN. Assessment of catheter ablation or antiarrhythmic drugs for first-line therapy of atrial fibrillation: a meta-analysis of randomized clinical trials. JAMA Cardiol. (2021) 6:697–705. 10.1001/jamacardio.2021.0852 33909022 PMC8082432

[17] Mark DB Anstrom KJ Sheng S Piccini JP Baloch KN Monahan KH . Effect of catheter ablation vs. medical therapy on quality of life among patients with atrial fibrillation: the CABANA randomized clinical trial. JAMA. (2019) 321:1275–85. 10.1001/jama.2019.0692 30874716 PMC6450275

[18] Diener HC Hart RG Koudstaal PJ Lane DA Lip GYH. Atrial fibrillation and cognitive function: JACC review topic of the week. J Am Coll Cardiol. (2019) 73:612–9. 10.1016/j.jacc.2018.10.077 30732716

[19] Poole JE Bahnson TD Monahan KH Johnson G Rostami H Silverstein AP . Recurrence of atrial fibrillation after catheter ablation or antiarrhythmic drug therapy in the CABANA trial. J Am Coll Cardiol. (2020) 75:3105–18. 10.1016/j.jacc.2020.04.065 32586583 PMC8064404

[20] Nakamura K Sasaki T Take Y Minami K Inoue M Asahina C . Incidence and characteristics of silent cerebral embolisms after radiofrequency-based atrial fibrillation ablation: a propensity score-matched analysis between different mapping catheters and indices for guiding ablation. J Cardiovasc Electrophysiol. (2021) 32:16–26. 10.1111/jce.14800 33141496

[21] Liberati A Altman DG Tetzlaff J Mulrow C Gøtzsche PC Ioannidis JPA . The PRISMA statement for reporting systematic reviews and meta-analyses of studies that evaluate health care interventions: explanation and elaboration. PLoS Med. (2009) 6:e1000100. 10.1371/journal.pmed.1000100 19621070 PMC2707010

[22] Margulis AV Pladevall M Riera-Guardia N Varas-Lorenzo C Hazell L Berkman ND . Quality assessment of observational studies in a drug-safety systematic review, comparison of two tools: the Newcastle-Ottawa scale and the RTI item bank. Clin Epidemiol. (2014) 6:359–68. 10.2147/CLEP.S66677 25336990 PMC4199858

[23] Guyatt G Oxman AD Akl EA Kunz R Vist G Brozek J . GRADE guidelines: 1. Introduction-GRADE evidence profiles and summary of findings tables. J Clin Epidemiol. (2011) 64:383–94. 10.1016/j.jclinepi.2010.04.026 21195583

[24] Kim D Yang PS Sung JH Jang E Yu HT Kim TH . Less dementia after catheter ablation for atrial fibrillation: a nationwide cohort study. Eur Heart J. (2020) 41:4483–93. 10.1093/eurheartj/ehaa726 33022705

[25] Bunch TJ Bair TL Crandall BG Cutler MJ Day JD Graves KG . Stroke and dementia risk in patients with and without atrial fibrillation and carotid arterial disease. Heart Rhythm. (2020) 17:20–6. 10.1016/j.hrthm.2019.07.007 31299299

[26] Hsieh YC Chen YY Chien KL Chung FP Lo LW Chang SL . Catheter ablation of atrial fibrillation reduces the risk of dementia and hospitalization during a very long-term follow-up. Int J Cardiol. (2020) 304:75–81. 10.1016/j.ijcard.2019.12.016 31884008

[27] Bunch TJ Crandall BG Weiss JP May HT Bair TL Osborn JS . Patients treated with catheter ablation for atrial fibrillation have long-term rates of death, stroke, and dementia similar to patients without atrial fibrillation. J Cardiovasc Electrophysiol. (2011) 22:839–45. 10.1111/j.1540-8167.2011.02035.x 21410581

[28] Tischer TS Nitschke D Krause I Öner A D'Ancona G Safak E . Prevalence and progression of cognitive impairment in atrial fibrillation patients after treatment with catheter ablation or drug therapy. Cardiol Res Pract. (2019) 2019:7216598. 10.1155/2019/7216598 31915546 PMC6931025

[29] Jin MN Kim TH Kang KW Yu HT Uhm JS Joung B . Atrial fibrillation catheter ablation improves 1-year follow-up cognitive function, especially in patients with impaired cognitive function. Circ Arrhythm Electrophysiol. (2019) 12:e007197. 10.1161/CIRCEP.119.007197 31442075

[30] Huang JT Tan Y Yang BP. Effects of anticoagulation and radiofrequency ablation on cognitive function in patients with atrial fibrillation. Clin Neurol J. (2021) 34:165–8. 10.3969/j.issn.1004-1648.2021.03.002

[31] Xia CC. Effects of Radiofrequency Ablation on Cognitive Function of Patients With Atrial Fibrillation. Changsha: Central South University (2014).

[32] Wang X Wang Z Yan X Huang M Wu Y. Radiofrequency and cryoballoon ablation improve cognitive function in patients with atrial fibrillation. Medicine (Baltimore). (2021) 100:e26914. 10.1097/MD.0000000000026914 34397930 PMC8360464

[33] Medi C Evered L Silbert B The A Halloran K Morton J. Subtle post-procedural cognitive dysfunction after atrial fibrillation ablation. J Am Coll Cardiol. (2013) 62:531–9. 10.1016/j.jacc.2013.03.073 23684686

[34] Zhang J Xia SJ Du X Jiang C Lai YW Wang YF . Incidence and risk factors of post-operative cognitive decline after ablation for atrial fibrillation. BMC Cardiovasc Disord. (2021) 21:341. 10.1186/s12872-021-02139-7 34261448 PMC8278748

[35] Li F. Comparison in Quality of Life and Cognitive Function Before and After Radiofrequency Ablation and Cryoablation in Patients with Atrial Fibrillation. Dalian: Dalian Medical University (2020).

[36] Kato N Muraga K Hirata Y. Brain magnetic resonance imaging and cognitive alterations after ablation in patients with atrial fibrillation. Sci Rep. (2021) 11:18995. 10.1038/s41598-021-98484-w 34556757 PMC8460624

[37] Schwarz N Kuniss M Nedelmann M Kaps M Bachmann G Neumann T . Neuropsychological decline after catheter ablation of atrial fibrillation. Heart Rhythm. (2010) 7:1761–7. 10.1016/j.hrthm.2010.07.035 20691284

[38] Deneke T Shin DI Balta O Bünz K Fassbender F Mügge A . Post ablation asymptomatic cerebral lesions: long-term follow-up using magnetic resonance imaging. Heart Rhythm. (2011) 8:1705–11. 10.1016/j.hrthm.2011.06.030 21726519

[39] Bellmann B Fiebach JB Guttmann S Lin T Haeusler KG Bathe-Peters R . Incidence of MRI-detected brain lesions and neurocognitive function after electrical cardioversion in anticoagulated patients with persistent atrial fibrillation. Int J Cardiol. (2017) 243:239–43. 10.1016/j.ijcard.2017.05.102 28592382

[40] Nakamura K Naito S Sasaki T Minami K Take Y Goto E . Silent cerebral ischemic lesions after catheter ablation of atrial fibrillation in patients on 5 types of periprocedural oral anticoagulation-predictors of diffusion-weighted imaging-positive lesions and follow-up magnetic resonance imaging. Circ J. (2016) 80:870–7. 10.1253/circj.CJ-15-1368 26888266

[41] Rillig A Meyerfeldt U Tilz RR Talazko J Arya A Zvereva V . Incidence and long-term follow-up of silent cerebral lesions after pulmonary vein isolation using a remote robotic navigation system as compared with manual ablation. Circ Arrhythm Electrophysiol. (2012) 5:15–21. 10.1161/CIRCEP.111.967497 22247481

[42] Rosman L Burg MM Lampert R. Catheter ablation and cognitive impairment in atrial fibrillation: another hit or a silver bullet? Circ Arrhythm Electrophysiol. (2019) 12:e007521. 10.1161/CIRCEP.119.007521 31442073 PMC7384245

[43] Chinta V Askandar S Nanda A Sharma A Abader P Kabra R . Atrial fibrillation and deterioration in cognitive function. Curr Probl Cardiol. (2018) 44:100386. 10.1016/j.cpcardiol.2018.07.001 30193747

[44] Madhavan M Graff-Radford J Piccini JP Gersh BJ. Cognitive dysfunction in atrial fibrillation. Nat Rev Cardiol. (2018) 15:744–56. 10.1038/s41569-018-0075-z 30275499

[45] Dietzel J Haeusler KG Endres M. Does atrial fibrillation cause cognitive decline and dementia? Europace. (2018) 20:408–19. 10.1093/europace/eux031 28387847

[46] Kim D Yang PS You SC Sung JH Jang E Yu HT . Association of rhythm control with incident dementia among patients with atrial fibrillation: a nationwide population-based cohort study. Age Ageing. (2022) 51:afab248. 10.1093/ageing/afab248 35061873

[47] Haeusler KG Eichner FA Heuschmann PU Fiebach JB Engelhorn T Blank B . MRI-detected brain lesions and cognitive function in patients with atrial fibrillation undergoing left atrial catheter ablation in the randomized AXAFA-AFNET 5 trial. Circulation. (2022) 145:906–15. 10.1161/CIRCULATIONAHA.121.056320 35135308

[48] Saglietto A Ballatore A Xhakupi H De Ferrari GM Anselmino M. Association of catheter ablation and reduced incidence of dementia among patients with atrial fibrillation during long-term follow-up: a systematic review and meta-analysis of observational studies. J Cardiovasc Dev Dis. (2022) 9:140. 10.3390/jcdd9050140 35621851 PMC9143892

[49] Bodagh N Yap R Kotadia I Sim I Bhalla A Somerville P . Impact of catheter ablation versus medical therapy on cognitive function in atrial fibrillation: a systematic review. J Interv Card Electrophysiol. (2022). 10.1007/s10840-022-01196-y [Epub ahead of print]. 35380337 PMC9550702

