# Supplementary material for: Catheter ablation and cognitive function in atrial fibrillation: A systematic review and meta-analysis
Source: Front Neurol. 2022 Sep 8;13:934512. doi: 10.3389/fneur.2022.934512 (PMC9492882; doi:10.3389/fneur.2022.934512)
Supplement: Supplementary file 1 [file Data_Sheet_1.doc]

**Full Search Strategy and Results**

| **Database**  **(Search Date)** | **Search Terms** | **Filters** | **Number of results** |
| --- | --- | --- | --- |
| Pubmed  (28/9/2021) | #1 Atrial Fibrillation[mh] or Fibrillation*, Atrial[tiab] or Auricular Fibrillation*[tiab] or Persistent Atrial Fibrillation*[tiab] or Paroxysmal Atrial Fibrillation*[tiab] or Familial Atrial Fibrillation*[tiab]#2 Catheter Ablation[mh] or Cryosurgery[mh] or Radiofrequency Ablation[mh] or Ablation, Catheter[tiab] or Transvenous Catheter Ablation[tiab] or Electrical* Catheter Ablation[tiab] or Transvenous Electric* Ablation[tiab] or Percutaneous Catheter Ablation[tiab] or Cryosurgeries[tiab] or Cryoablation*[tiab] #3 Dementia[mh] or Dementia, Vascular[mh] or Alzheimer Diseaseor[mh] or Amentia[tiab] or Familial Dementia*[tiab] or Vascular Dementia*[tiab] or Subcortical Vascular Dementia*[tiab] or Alzheimer Dementia*[tiab] or Senile Dementia[tiab] or Alzheimer Sclerosis[tiab] or Alzheimer Syndrome[tiab] or Alzheimer* Diseases[tiab] #4 Cognitive Dysfunction[mh] or Cognition Disorders[mh] or Cognitive Dysfunctions*[tiab] or Cognitive Impairment*[tiab] or Mild Neurocognitive Disorder*[tiab] or Cognitive Decline*[tiab] or Mental Deterioration*[tiab] #5 Mental Status and Dementia Tests[mh] or General Practitioner Assessment of Cognition[tiab] or Montreal Cognitive Assessment[tiab] or Mental Status Tests*[tiab] or Neurocognitive Tests[tiab] or Mini Mental State Examination[tiab] or Clinical Dementia Rating[tiab] or Telephone Interview for Cognitive Status-modified[tiab] #6 #3 OR #4 OR #5#7 #1 AND #2 AND #6 | Language: English | 116 |
| Embase (28/9/2021) | #1 (Atrial Fibrillation).exp.#2 (Persistent Atrial Fibrillation).ti,ab.#3 (Paroxysmal Atrial Fibrillation).ti,ab.#4 ( Auricular Fibrillation).ti,ab.#5 #1 OR #2 OR #3 OR #4#6 (Catheter Ablation).exp.#7 (Cryosurgery*).exp.#8 (Radiofrequency Ablation).exp.#9 (Transvenous Catheter Ablation).mp.#10 (Electrical Catheter Ablation).mp.#11 (Percutaneous Catheter Ablation).mp.#12 (Cryosurgeries*).mp.#13 (Cryoablation*).mp.#14 #5 OR #6 OR #7 OR #8 OR #9 OR #10 OR #11 OR #12 OR #13 #15 (Dementia).exp.#16 (Vascular Dementia,).exp.#17 (Alzheimer Diseaseor).exp.#18 (Senile Dementia).ti,ab.#19 (Familial Dementia).ti,ab.#20 (Subcortical Vascular Dementia).ti,ab.#21 (Alzheimer Dementia).ti,ab.#22 (Alzheimer Sclerosis).ti,ab.#23 (Alzheimer Syndrome).ti,ab.#24 (Alzheimer Diseases).ti,ab.#25 (Cognitive Dysfunction).exp.#26 (Cognition Disorders).exp.#27 (Cognitive Impairment).ti,ab.#28 (Mild Neurocognitive Disorder).ti,ab.#29 (Mental Deterioration).ti,ab.#30 (Cognitive Decline).ti,ab.#31 (Mental Status and Dementia Tests).exp.#32 (General Practitioner Assessment of Cognition).ti,ab.#33 (Montreal Cognitive Assessment).ti,ab.#34 (Mental Status Tests).ti,ab.#35 (Neurocognitive Tests).ti,ab.#36 (Mini Mental State Examination).ti,ab.#37 (Clinical Dementia Rating).ti,ab.#38 (Telephone Interview for Cognitive Status-modified).ti,ab.#39 #15 OR #16 OR #17 OR #18 OR #19 OR #20 OR #21 OR #22 OR #23 OR #24 OR #25 OR #26 OR #27 OR #28 OR #29 OR #30 OR #31 OR #32 OR #33 OR #34 OR #35 OR #36 OR #37 OR #38#40 #5 AND #14 AND #39 | Language: English | 214 |
| Web of science  (28/9/2021) | #1(((TS=(Atrial Fibrillation)) OR TS=(Auricular Fibrillation)) OR TS=(Persistent Atrial Fibrillation)) OR TS=(Paroxysmal Atrial Fibrillation)  Databases=SCI-EXPANDED, SSCI, A&HCI, CPCI-S, CPCI-SSH, BKCI-S, BKCI-SSH, ESCI, CCR-EXPANDED, IC Timespan= 1970-2021#2(((((((TS=(Catheter Ablation)) OR TS=(Cryosurgery*)) OR TS=(Radiofrequency Ablation)) OR TS=(Transvenous Catheter Ablation)) OR TS=(Electrical* Catheter Ablation)) OR TS=(Percutaneous Catheter Ablation)) OR TS=(Cryosurgeries*)) OR TS=(Cryoablation*)  Databases=SCI-EXPANDED, SSCI, A&HCI, CPCI-S, CPCI-SSH, BKCI-S, BKCI-SSH, ESCI, CCR-EXPANDED, IC Timespan= 1970-2021#3(((((((((TS=(Dementia)) OR TS=(Alzheimer Diseaseor)) OR TS=(Familial Dementia)) OR TS=(Vascular Dementia)) OR TS=(Subcortical Vascular Dementia)) OR TS=(Alzheimer Dementia)) OR TS=(Senile Dementia)) OR TS=(Alzheimer Sclerosis)) OR TS=(Alzheimer Syndrome)) OR TS=(Alzheimer Diseases)  Databases=SCI-EXPANDED, SSCI, A&HCI, CPCI-S, CPCI-SSH, BKCI-S, BKCI-SSH, ESCI, CCR-EXPANDED, IC Timespan= 1970-2021#4((((((TS=(Cognitive Dysfunction)) OR TS=(Cognition Disorders)) OR TS=(Cognitive Dysfunctions)) OR TS=(Cognitive Impairment)) OR TS=(Mild Neurocognitive Disorder)) OR TS=( Cognitive Decline)) OR TS=(Mental Deterioration)  Databases=SCI-EXPANDED, SSCI, A&HCI, CPCI-S, CPCI-SSH, BKCI-S, BKCI-SSH, ESCI, CCR-EXPANDED, IC Timespan= 1970-2021#5(((((((TS=(Mental Status and Dementia Tests)) OR TS=(General Practitioner Assessment of Cognition)) OR TS=(Montreal Cognitive Assessment)) OR TS=(Mental Status Tests)) OR TS=(Neurocognitive Tests)) OR TS=(Mini Mental State Examination)) OR TS=(Clinical Dementia Rating)) OR TS=(Telephone Interview for Cognitive Status-modified)  Databases=SCI-EXPANDED, SSCI, A&HCI, CPCI-S, CPCI-SSH, BKCI-S, BKCI-SSH, ESCI, CCR-EXPANDED, IC Timespan= 1970-2021#6 #3 OR #4 OR #5#7 #1 AND #2 AND #6 | Language: English | 147 |
| CNKI, Wanfang and VIP databases(28/9/2021) | ((TKA:Atrial Fibrillation) OR (TKA:Persistent Atrial Fibrillation) OR (TKA:Paroxysmal Atrial Fibrillation) OR (TKA:Familial Atrial Fibrillation)) AND ((TKA:Catheter Ablation) OR (TKA:Radiofrequency Ablation) OR (TKA:Transvenous Catheter Ablation) OR (TKA:Percutaneous Catheter Ablation) OR (TKA:Electrical Catheter Ablation) OR (TKA:Cryoablation)) AND ((TKA:Dementia) OR (TKA:Alzheimer Diseaseor) OR (TKA:Vascular Dementia) OR (TKA:Familial Dementia) OR (TKA:Cognitive Dysfunction) OR (TKA:Cognitive Decline) OR (TKA:Mental Deterioration) OR (TKA:Mental Status) OR (TKA:Dementia Tests) OR (TKA:Montreal Cognitive Assessment) OR (TKA:Mental Status Tests) OR (TKA:Mini Mental State Examination) OR (TKA:Telephone Interview for Cognitive Status-modified) OR (TKA:cognitive function scale)) | Language:  No  restriction | CNKI:65 WanFang:71VIP:42 |

**The Exclusion Studies and Reason**

| **Reason for Exclusion** | **Study Name** | **Reference** |
| --- | --- | --- |
| Review | Rosman L et al 2019 | Rosman L, Burg MM, Lampert R. Catheter Ablation and Cognitive Impairment in Atrial Fibrillation: Another Hit or a Silver Bullet? Circ Arrhythm Electrophysiol. 2019;12(7):e007521. doi: 10.1161/CIRCEP.119.007521. |
| Review | Camm AJ et al 2020 | Camm AJ. Does ablation of atrial fibrillation reduce the likelihood of dementia? A step closer but not yet there. Eur Heart J. 2020;41(47):4494-4496. doi: 10.1093/eurheartj/ehaa843. |
| Review | Healey JS et al 2020 | Healey JS, Nair GM. Does catheter ablation for atrial fibrillation increase or reduce neurological insult? Curr Opin Cardiol. 2012;27(1):36-40. doi: 10.1097/HCO.0b013e32834d846a. |
| Not report the cognitive outcomes | Bellmann B et al 2017 | Bellmann B, Fiebach JB, Guttmann S, et al. Incidence of MRI-detected brain lesions and neurocognitive function after electrical cardioversion in anticoagulated patients with persistent atrial fibrillation. Int J Cardiol. 2017;243:239-243. doi: 10.1016/j.ijcard.2017.05.102. |
| Not report the cognitive outcomes | Forleo GB et al 2016 | Forleo GB, Della Rocca DG, Lavalle C, et al. A Patient With Asymptomatic Cerebral Lesions During AF Ablation: How Much Should We Worry? J Atr Fibrillation. 2016;8(5):1323. doi: 10.4022/jafib.1323. |
| Not report the cognitive outcomes | Kochhäuser S et al 2015 | Kochhäuser S, Lohmann HH, Ritter MA, et al. Neuropsychological impact of cerebral microemboli in ablation of atrial fibrillation. Clin Res Cardiol. 2015 ;104(3):234-40. doi: 10.1007/s00392-014-0777-0. |
| Not report the cognitive outcomes | Schwarz N et al 2010 | Schwarz N, Kuniss M, Nedelmann M, et al. Neuropsychological decline after catheter ablation of atrial fibrillation. Heart Rhythm. 2010;7(12):1761-7. doi: 10.1016/j.hrthm.2010.07.035. |
| Not report the cognitive outcomes | Patel D et al 2009 | Patel D, Bailey SM, Furlan AJ, et al. Long-term functional and neurocognitive recovery in patients who had an acute cerebrovascular event secondary to catheter ablation for atrial fibrillation. J Cardiovasc Electrophysiol. 2010;21(4):412-7. doi: 10.1111/j.1540-8167.2009.01650.x. Epub 2009 Nov 17. |
| Not report the cognitive outcomes | Hyogo K et al 2019 | Hyogo K, Yoshida A, Takeuchi M, et al. One-year clinical outcomes of anticoagulation therapy among Japanese patients with atrial fibrillation: The Hyogo AF Network (HAF-NET) Registry. J Arrhythm. 2019;35(5):697-708. doi: 10.1002/joa3.12226. |
| Not report the cognitive outcomes | Schmidt B et al 2019 | Schmidt B, Széplaki G, Merkely B, et al. Silent cerebral lesions and cognitive function after pulmonary vein isolation with an irrigated gold-tip catheter: REDUCE-TE Pilot study. J Cardiovasc Electrophysiol. 2019 ;30(6):877-885. doi: 10.1111/jce.13902. |
| Incomplete data study | Herm J et al 2020 | Herm J, Schirdewan A, Koch L,et al. Impact of atrial fibrillation burden on cognitive function after left atrial ablation - Results of the MACPAF study. J Clin Neurosci. 2020;73:168-172. doi: 10.1016/j.jocn.2019.12.030. |
| Incomplete data study | Kim D et al 2020 | Kim D, Yang PS, You SC, et al. Association of rhythm control with incident dementia among patients with atrial fibrillation: a nationwide population-based cohort study. Age Ageing. 2022;51(1):afab248. doi: 10.1093/ageing/afab248. |
| Incomplete data study | Kloosterman M et al 2020 | Kloosterman M, Chua W, Fabritz L,et al. Sex differences in catheter ablation of atrial fibrillation: results from AXAFA-AFNET 5. Europace. 2020;22(7):1026-1035. doi: 10.1093/europace/euaa015. |
